# Blended Mobile-Based Interventions With Integrated Virtual Reality Exposure Therapy for Anxiety Disorders: Thematic Analysis of Patient Perspectives

**DOI:** 10.2196/60957

**Published:** 2025-04-24

**Authors:** Jari Planert, Anne-Sophie Hildebrand, Alla Machulska, Kati Roesmann, Marie Neubert, Sebastian Pilgramm, Juliane Pilgramm, Tim Klucken

**Affiliations:** 1 Department of Psychology Clinical Psychology and Psychotherapy University of Siegen Siegen Germany; 2 Institute of Psychology Clinical Psychology and Psychotherapy in Childhood and Adolescence University of Osnabrück Osnabrück Germany; 3 Department of Psychology IU International University of Applied Sciences Erfurt Germany

**Keywords:** virtual reality exposure therapy, anxiety disorders, internet- and mobile-based interventions, blended therapy, eHealth

## Abstract

**Background:**

Guided mobile-based interventions may mitigate symptoms of anxiety disorders such as panic disorder, agoraphobia, or social anxiety disorder. With exposure therapy being efficacious in traditional treatments for these disorders, recent advancements have introduced 360° videos to deliver virtual reality exposure therapy (VRET) within mobile-based interventions.

**Objective:**

Despite ongoing trials evaluating the treatment’s efficacy, research examining patient perceptions of this innovative approach is still scarce. Therefore, this study aimed to explore patient opinions on specific treatment aspects of mobile-based interventions using mobile VRET and psychotherapeutic guidance for anxiety disorders.

**Methods:**

A total of 11 patients diagnosed with panic disorder, agoraphobia, or social anxiety disorder who had previously taken part in the experimental conditions of 2 randomized controlled trials for a mobile intervention including mobile VRET participated in cross-sectional, retrospective interviews. Using a semistructured interview format, patients were asked to reflect on their treatment experiences; personal changes; helpful and hindering aspects; their motivation levels; and their encounters with the mobile-based intervention, manualized treatment sessions, and the mobile VRET.

**Results:**

Thematic analysis led to the formation of 14 themes in four superordinate categories: (1) perceived treatment outcomes, (2) aspects of the mobile intervention, (3) experiences with mobile VRET, and (4) contextual considerations. Patients offered their insights into factors contributing to treatment success or failure, delineated perceived treatment outcomes, and highlighted favorable aspects of the treatment while pointing out shortcomings and suggesting potential enhancements. Most strikingly, while using a blended app-based intervention, patients highlighted the role of psychotherapeutic guidance as a central contributing factor to their symptom improvement.

**Conclusions:**

The findings of the thematic analysis and its diverse patient perspectives hold the potential to guide future research to improve mobile-based treatment options for anxiety disorders. Insights from these patient experiences can contribute to refining mobile-based interventions and optimizing the integration of VRET in accordance with patients’ preferences, needs, and expectations.

## Introduction

### Background

Anxiety disorders such as social anxiety disorder (SAD), panic disorder (PD), or agoraphobia (AG) are a pervasive public health issue with 12-month prevalence rates between 0.9% and 1.2%, causing severe impairment for those affected [1-3]. SAD, PD, and AG are characterized by intense fear reactions toward specific situations or bodily sensations that are normally perceived as harmless or nonthreatening [4]. In SAD, typical anxiety-provoking situations include social interactions (eg, being at the center of attention and meeting new people) as the fear is linked to negative evaluation, criticism, or embarrassment and rejection [5]. In PD, panic attacks are triggered by an overinterpretation of occasional bodily arousal (eg, elevated heartbeat) as life-threatening (eg, having a myocardial infarction) [6]. The catastrophic misinterpretation of such sensations can then lead individuals to be afraid of losing control or experience fear of death. In contrast, AG entails paniclike fear in situations in which escape is difficult (shopping queues, the subway, or car rides) as patients often describe their main concern revolving around losing control, having a medical emergency, being trapped, or not receiving help [7]. Affected individuals often describe anticipatory anxiety as especially troublesome, which may explain why all 3 disorders are often associated with avoidance and safety behavior [8,9].

Cognitive behavioral therapy (CBT) has been found highly effective in treating PD and AG. [10]. Typical methods used in CBT for these disorders are psychoeducation, behavioral experiments, and exposure therapy [11]. However, despite compelling evidence supporting the treatment’s efficacy [12], most affected individuals fail to receive treatment (ie, according to Stein et al [13], less than a quarter of patients are admitted for treatment). One reason that keeps individuals in need from receiving treatment is the increasingly exhausted supply capabilities in mental health care, leading to prolonged waiting periods and large pretreatment attrition rates [14]. This is further aggravated by the fact that treatment recommendations such as exposure therapy often remain unfollowed due to time constraints, high costs, and resource demands [15,16].

Exposure therapy, one of the most effective treatments for anxiety disorders, involves the systematic confrontation with feared stimuli or situations to reduce avoidance behavior and facilitate fear extinction. There are several underlying mechanisms that may explain the effect of exposure therapy. First, early theories emphasize that repeated confrontation with anxiety-inducing stimuli promotes habituation [17], leading to a reduction in fear responses and supporting a learning process whereby patients experience that anxiety can diminish without avoidance. Second, confrontation also addresses general therapeutic factors such as problem activation and problem-solving, which have been repeatedly shown to be key mechanisms in psychotherapy [18]. Current theories suggest that the central mechanism in exposure therapy is inhibitory learning, which is induced by expectancy violations. In this framework, to optimize exposure success, it is key to challenge patients’ predictions about feared outcomes [19,20]. Despite the documented efficacy and repeated validation of exposure therapy, it remains underused in clinical practice. A contributing factor is the underdiagnosis of anxiety disorders, which reduces access to appropriate treatment [21,22]. However, even when correctly diagnosed, the implementation of exposure therapy is not guaranteed. Reasons for this vary, including logistical challenges, a lack of resources, concerns about patient distress, or insufficient training [16]. In addition, patients might be reluctant to confront feared stimuli or discontinue treatment, which poses additional considerable barriers.

A recent approach to bridge the shortage of treatment capabilities in mental health care is using internet- and mobile-based interventions (IMIs) [23], which include smartphone apps that are tailored for specific psychological disorders such as SAD, PD, and AG. Most of these apps incorporate basic CBT interventions and have been shown to be effective in reducing symptoms, for example, through (a combination of) psychoeducation, cognitive restructuring, mindfulness, and relaxation techniques [24-26]. IMIs can follow a stand-alone approach or may also include varying degrees of psychotherapeutic guidance. Regarding the latter, guided treatment programs have been shown to be superior to stand-alone approaches in reducing clinical symptoms, as indicated by larger acceptability, engagement, and adherence [23,26]. These factors are crucial as they have been linked to improved treatment outcomes and sustained recovery [26]. Possible advantages of IMIs include flexible use at almost any time and place and relieving the psychotherapists’ time burden [27]. While the use of IMIs could constitute an option to bridge scarce treatment capabilities, little is known about the potential of IMIs with exposure components to overcome these boundaries and provide less resource-intensive and more accessible exposure therapy as the first-line treatment option for anxiety disorders [28]. Therefore, the question arises of how exposure therapy can be integrated within IMIs.

One way to reduce barriers in the delivery of exposure therapy is the transfer of anxiety-eliciting situations to a virtual reality (VR) environment. VR exposure therapy (VRET) allows for the creation of specific scenarios that may be challenging or impractical to replicate in real life (in vivo) and that are tailored to elicit fear responses [29-31]. These simulations can be adjusted according to each patient’s unique needs (ie, massive or gradual approach) and specific concerns while therapists maintain full control during such sessions [32,33]. For instance, therapists can pause, repeat, or pick scenarios based on the patients’ progress, feedback, or observed reactions, whereas no unforeseeable events can appear as in in vivo therapy. Even if a certain level of risk is necessary for successful exposure therapy, which cannot be guaranteed by the artificial nature of VRET, the so-called immersion (ie, the feeling of realism created in VR atmospheres) helps uphold the patients’ apprehension of imminent danger. This, in turn, can activate central fears and allow for their correction. Moreover, aversive risks such as physical harm can mostly be ruled out in VRET, rendering it to a certain degree safer than in vivo exposure therapy. So far, VRET has been used in the treatment of multiple anxiety disorders, such as SAD or AG, to reduce symptoms and improve quality of life successfully [34,35]. For instance, patients with SAD have experienced significant reductions in fear of public speaking or social interactions [36-39]. Patients with AG have had less anxiety and shown less avoidance when traveling or enduring crowded places [40]. The quantity and duration of VRET sessions varies in the literature, typically lasting between 30 and 60 minutes and being conducted 8 to 12 times. Compared to exposure in vivo, increased acceptance and lower refusal rates have been reported in the past, making VRET a low-cost treatment option with a high success rate [30,41].

VRET has been used in mainly 2 ways in the existing literature. The first involves a computer-based system, allowing patients to engage with avatars, affect their surroundings, or receive immediate feedback during exposure [42]. Advantages of this approach include a high degree of immersion, the ability to tailor scenarios to individual needs, and the option to simulate dynamic (eg, social) interactions. The second approach uses 360° video systems, which present prerecorded scenarios without the possibility of affecting these through interaction. Notable advantages of this method are low costs, effortless scalability, and integrability with other mental health approaches such as IMIs as it requires considerably less technical equipment than interactive VR systems [43]. However, theoretical models of SAD [5] suggest that approaches with higher degrees of interaction might be more beneficial as fear is linked to interpersonal feedback or interaction [44]. Interestingly, both approaches have been found efficacious in reducing symptoms of SAD, PD, and AG [45]. However, research that directly compares interactive VRET systems and VRET via 360° video systems for the treatment of the aforementioned disorders is still scarce.

Taken together, the use of an IMI might reduce waiting times, whereas VRET might help implement exposure without high costs and resources [30]. To benefit from and provide additional advantages from the combined use, new approaches most recently have tried to integrate mobile VRET into IMIs [46-48]. Instead of using expensive VR equipment, patients use a head-mounted construction in which they can insert their smartphone as a screen and display 360° videos. By doing so, it has been demonstrated that treating acrophobia via self-guided VRET is possible [46,49]. Currently, randomized controlled trials (RCTs) are ongoing to investigate the efficacy of improving symptoms of SAD, PD, and AG using this innovative treatment approach [47,48,50].

To date, compared to a robust body of quantitative research, there are considerably less qualitative studies that have examined VRET. Research investigating the patient perspective has found that immersion and engagement are critical factors for its success, with patients highly valuing the ability to confront fears in a controlled and virtual environment [51,52]. However, perceived challenges are limited interactivity in prerecorded scenarios and technical difficulties, which may decrease the immersive experience and, therefore, the perceived effectiveness of the intervention [51,52]. Regarding the use of IMIs, qualitative research on patients’ opinions has highlighted high accessibility and flexibility as major strengths while raising concerns about the lack of relational and emotional support inherent to the oftentimes self-guided nature of IMIs [53,54]. Although the cited literature provides valuable insights into the perspective of patients using VRET and IMIs separately, qualitative research regarding the integration of mobile VRET within guided IMIs is scarce. This research gap is addressed in this study.

### Objectives

This study aimed to investigate patients’ perspectives on integrating mobile VRET into guided IMIs for SAD, PD, and AG. Specifically, the goal was to identify key aspects that patients perceive as relevant to improvement or deterioration, including factors associated with the treatment’s limitations, benefits, and outcomes [55,56]. This study also attempted to explore how patients navigate through the different treatment components, what their subjective reasons were for experiencing change or not, and their views on the combination of app-based treatment with psychotherapeutic guidance. On this basis, ideas can be formed on how to improve further similar approaches by facilitating patient adherence.

## Methods

### Participants

Patients were recruited from 2 RCTs that used a blended treatment approach comprising an IMI with in-app mobile VRET and appointments with a licensed psychotherapist. The work by Planert et al [47] and Hildebrand et al [48] provides further details concerning the trials.

Inclusion criteria were (1) fulfilling the *International Classification of Diseases, 10th Revision,* diagnostic criteria for one of the following psychological disorders: *agoraphobia, unspecified* (F40.00), *agoraphobia with panic disorder* (F40.01), *agoraphobia without panic disorder* (F40.02), *social phobia* (F40.1), or *panic disorder* (F41.0); (2) being aged ≥18 years; and (3) having completed the treatment in one of the RCTs [47,48]. Exclusion criteria for participation in the trials included a history of cardiovascular conditions (eg, stroke or myocardial infarction), respiratory disorders (eg, asthma or chronic obstructive pulmonary disease), epilepsy, pregnancy, impaired vision, schizophrenia, severe depression, acute suicidality, or substance use disorders. None of the patients received outpatient psychotherapy during the treatment period apart from the therapy provided as part of the study. Only participants from the treatment group in either of the 2 trials were invited upon completion of the RCT. A total of 11 patients (n=5, 45% patients with SAD; n=6, 55% patients with PD or AG) agreed to partake in the interviews.

### Mobile-Based Intervention

The IMI in this blended treatment was an app for mobile phones incorporating 3 distinct treatment programs specifically developed for SAD, PD, and AG (Invirto [57]), of which patients completed 1. The app is based on a CBT treatment and consists of structured modules targeting different treatment aspects: goal setting, motivation, psychoeducation, tension regulation, cognitive techniques, exposure therapy, behavioral experiments, emotion regulation, exposure, reflection, and relapse prevention. For example, tension regulation was provided via breathing exercises, incorporating mindfulness-based techniques to help patients manage physiological arousal. Unlike classic behavioral therapy methods, which rely heavily on face-to-face interactions, the app enabled patients to work autonomously through the modules. While the psychotherapists provided some guidance during scheduled appointments, the intervention allowed patients to progress within the structured program at their own pace. Modules incorporated a mix of prerecorded video sequences of psychotherapists, interactive experiments, self-assessments, exercises, and a wide range of potentially fear-evoking situations in a controlled VR environment. Using 360° videos, patients were able to choose scenarios such as giving a public speech, undergoing a job interview, or being in a crowded space or public transport that were thought to provoke or aimed at provoking symptoms of their specific anxiety disorder.

### Procedure

**Figure 1:**
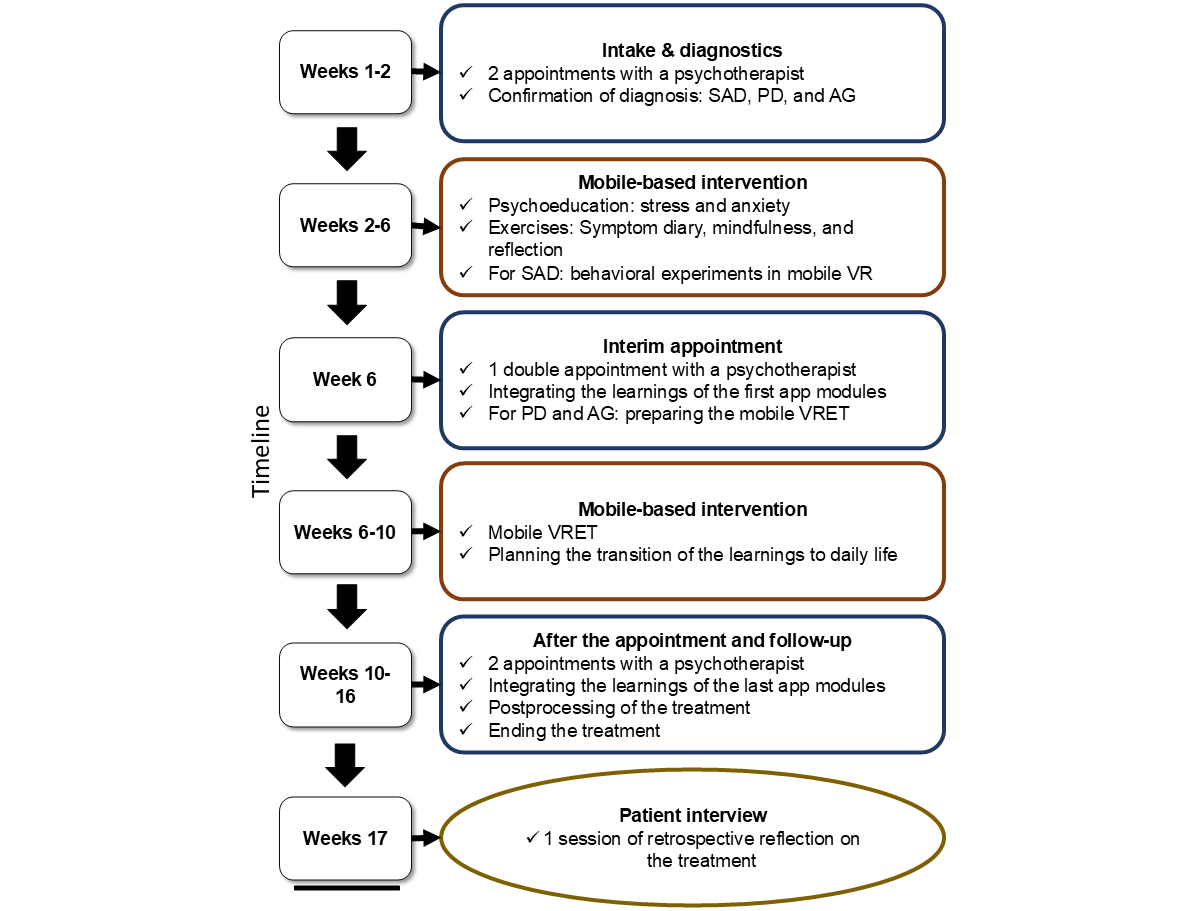
Procedure of the treatment process and study schedule. On the left, the boxes indicate the corresponding week of the trial. Blue boxes indicate psychotherapeutic sessions, whereas the red boxes indicate periods in which the patients worked independently with the treatment app. The yellow box marks the time point at which the patient interviews took place. AG: agoraphobia; PD: panic disorder; SAD: social anxiety disorder; VR: virtual reality; VRET: virtual reality exposure therapy.

Throughout the blended treatment, 5 psychotherapeutic appointments were conducted (for details, see the work by Planert et al [47] and Hildebrand et al [48]). As the mobile-based app offers 3 different treatment programs depending on the anxiety disorder, a joint recruitment process took place. Figure 1 provides an overview of the treatment procedure and study schedule.

At study entry, patients received 2 diagnostic appointments in which they were assessed for eligibility. During these appointments, the diagnosis was checked, and an anamnesis took place. Those diagnosed with PD, AG, or SAD received access to the app (ie, 1 of the 3 treatment programs), which was available for 90 days after prescription. They were instructed to complete the first modules (self-guided phase I), including psychoeducation on the corresponding disorder and exercises (eg, identifying and visualizing triggers of fear and exercising diaphragmatic breathing for PD to reduce hyperventilation and promote relaxation [58]). To check on the completion of the self-guided phase I, patients were called to check up on their progress and whether they were ready for the next appointment. The self-guided phase I was set to 6 weeks; however, progressing to the next appointment was only possible after completing all modules from phase I.

After completing the self-guided phase I, there was a third appointment to evaluate what patients had learned in the last modules and prepare for the next phase. As the treatment programs slightly differed with respect to the specific diagnosis, the content of the third appointment differed as well. For patients with SAD, the third appointment included evaluating the previous psychoeducation, exercises, and the behavioral experiments in mobile VR. In addition, the patients were prepared for the transfer of those behavioral experiments into everyday life. For patients with PD or AG, the evaluation included the previous psychoeducation and exercises without VR. The patients were prepared for behavioral exercises (eg, running upstairs) and exposure to mobile VR (eg, sitting in a car or train) via 360° videos without the possibility of interacting (eg, by provoking reactions from objects or persons nearby). Patients received additional guidance to apply previously learned breathing techniques and integrate these during exposure tasks, ensuring that patients were able to handle physiological symptoms of panic that would arise. After the third appointment, all patients had 4 weeks to complete the remaining modules (self-guided phase II). This included exposure in vivo, emotion regulation, and relapse prevention in all treatment programs. For patients with PD and AG, this also included exposure in VR in multiple scenarios (eg, a car or plane ride or a supermarket). Afterward, patients and psychotherapists evaluated the complete intervention in a fourth appointment. After 6 weeks, patients had a follow-up appointment to assess long-term changes in symptoms and were asked to participate in a half-standardized interview. The obtained data were retrospective insights from patients who completed the whole treatment and were able to extensively use the app in their daily routine.

### Half-Standardized Interview Procedure

All interviews were held in German and took, on average, 30 minutes and 40 seconds (SD 6 min). The interviews were conducted by author JP, a clinical psychologist. After describing the interview’s purpose and how data protection was ensured, each interview began with an open-ended question, providing interviewees with an initial opportunity to freely discuss the aspects of the treatment they found most notable. This was done by asking the following: “How did you experience the treatment you were taking part in?” Afterward, the interviewer followed a semistructured manual based on the Client Change Interview [59]. The Client Change Interview establishes aspects that are potentially associated with a client’s change in psychotherapy, such as helpful and hindering aspects, personal or psychological changes they made, or attributions for these changes. In this study, the interview manual was expanded through specific questions about the app included in the study, along with the mobile VRET, the study design of the RCT, and the expected role of the specific treatment in mental health care. The interview was semistructured, and for every point that the interviewee brought up, the interviewer was instructed to ask follow-up questions to ensure an in-depth understanding. All the interviews were semantically transcribed by student assistants. The interview procedure was conducted under the supervision of researchers AM and TK to ensure consistency and adherence to ethical guidelines.

### Data Analysis

To analyze the interview data, a thematic content analysis was conducted according to Braun and Clarke [55,56]. The analysis was carried out by authors JP (first coder) and ASH (second coder), both clinical psychologists, who independently from one another reviewed the data. An inductive approach was chosen to generate codes directly from the transcripts of the interviews.

**Figure 2:**
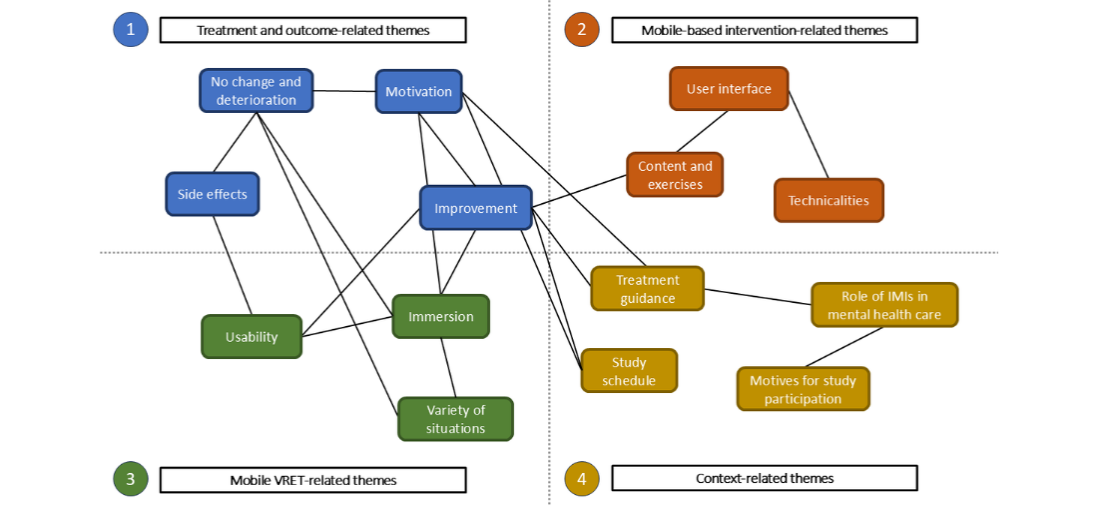
Thematic map of the established themes within their respective categories. Themes are divided by category. Links between the themes indicate thematic relatedness depending on the statements of the interviewed patients. IMI: internet- and mobile-based intervention; VRET: virtual reality exposure therapy.

The qualitative data analysis was conducted using MAXQDA 2022 (version 22.7.0; VERBI GmbH) [60]. As a first step, both coders intuitively went over a part of the interviews in a summarizing manner. This was done to establish codes representing the content of the interviews. At this point, codes were not structured. Afterward, the established codes were reviewed by both coders collaboratively to ensure accuracy and check on errors or parts and messages that the other coder might have missed. The generated codes were then interpreted and grouped into overarching categories (eg, VR-related content and IMI-related content) by 1 coder. The second coder then checked the categories and the assigned codes. Afterward, the codes in 1 overarching category were grouped into multiple themes by the second coder and checked by the first coder. Both coders then reflected on and interpreted the categories and themes with their corresponding codes and depicted them in a thematic map (Figure 2) to visually illustrate the findings.

### Ethical Considerations

The interview procedure after the follow-up appointment was approved by the Research Ethics Committee of the University of Siegen through an amendment to the corresponding ethics statement (ER_48_2021, amendment AZ_56/2023) All participants provided informed consent before participation, which could be withdrawn at any time without giving any reason. All collected data were pseudonymized to protect participants’ confidentiality. No monetary compensation was issued.

## Results

### Overview

As the interviews were conducted in German, extracted quotes were translated from German into English literally. The extracted quotes appear indented and in italics in the sections corresponding to each theme. Table 1 shows the demographic characteristics of the 11 interviewees.

**Table 1:** Demographic information of the study sample.

Patient code	Age range (y^a^)	Gender	Primary diagnosis (*ICD-10*^b^)	Symptom change^c^
P01	20-39	Woman	Social anxiety disorder	–8.5%
P02	20-39	Woman	Panic disorder	–50%
P03	20-39	Woman	Social anxiety disorder	–28.4%
P04	>40	Woman	Agoraphobia	–92.3%
P05	>40	Woman	Social anxiety disorder	–20.2%
P06	>40	Woman	Panic disorder	–73.2%
P07	>40	Woman	Panic disorder	–27.6%
P08	20-39	Woman	Agoraphobia	–70.1%
P09	>40	Man	Agoraphobia with panic disorder	–19.4%
P10	20-39	Woman	Social anxiety disorder	+10%
P11	20-39	Man	Social anxiety disorder	–3.8%

^a^Information on age is given as ranges to protect the anonymity of the interviewed patients.

^b^ *ICD-10: International Classification of Diseases, 10th Revision.*

^c^Symptom change indications refer to either of 2 disorder-specific questionnaires: the Questionnaire for Social Anxiety and Social Competence Deficits or the Panic and Agoraphobia Scale.

The themes of the various interview segments were divided into 4 overarching categories: “treatment and outcome–related themes,” “mobile-based intervention–related themes,” “VRET-related themes,” and “context-related themes” (Figure 2).

### Category 1: Treatment and Outcome–Related Themes

The first category covered themes that were related to patient-perceived aspects of the blended treatment or its outcomes. In total, this category consisted of 4 themes: symptom improvement, no change and symptom deterioration, side effects, and motivation throughout treatment.

#### Theme 1: Symptom Improvement

Throughout the treatment, many of the interviewed patients experienced symptom improvement. Indications as to how and why their symptoms might have improved are covered by this category’s first theme. While some patients reported a perceived symptom remission, others indicated that, while the treatment helped, they felt not fully recovered:

I was able to learn a lot and made big advancements. By now, I oftentimes go by car alone and I feel like I make progress every day, I also stopped thinking about my anxiety.

P04; position 8

Well, I still have anxiety. Partly, it got better.

P03; position 18

Various reasons were given for the perceived improvement. While some patients stressed the superior role of one component of the therapy (eg, therapeutic appointments, VRET, or psychoeducation), some pointed out that the comprehensive approach with a treatment app, mobile VRET, and psychotherapeutic appointments was helpful:

Yea, I’d say the therapist rather helped me, coming from an interpersonal understanding.

P02; position 29

It was an interplay. There was the app, in which I learned about anxiety and its causes. And then I had a person that accompanied or led me. The appointments helped me to keep going and push through.

P06; position 50

While sometimes the mobile VRET was highlighted as an especially helpful ingredient, others claimed that everything but the mobile VRET was beneficial:

And these experiments with the VR-headset. They really helped me, because in these situations, I really had anxiety, but I knew it was not real and it doesn’t matter how I behave.

P03; position 51

I think the video took an hour, I was on the highway and I was in the passenger seat. But being the passenger was no problem for me. That’s the same as if I was in somebody’s car, that doesn’t bother me either.

P09; position 54

In addition to the treatment components, symptom insight, learning to reflect on these symptoms, and receiving access to treatment after a long search were mentioned as crucial factors. Reflecting on their treatment progress, participants illuminated different protective factors that facilitated their symptom improvement, such as engaging modules, social support, hope, optimism, and motivation to use the app.

#### Theme 2: No Change and (Short-Term) Symptom Deterioration

Some patients reported retrospectively no change or even deterioration of their initial symptomatology. They brought up that the treatment was not able to address all aspects of their anxiety due to the manualization and the one-size-fits-all approach. In addition, they reported a lack of individualization and personalization:

It’s not possible as it is a pretty general format. For example, symptoms I don’t have are described or you are put in a state that does not cause anxiety. That is something I suspected to happen in the beginning.

P11; position 108

One patient brought up that, through psychoeducation, symptoms worsened by shifting the focus toward them:

I always watched the first few modules at night. This was usually the time when I got my panic attacks and listening to the modules was kind of a trigger for me. For example, it’s about what happens in your brain and what symptoms it can have. After hearing about the symptoms, I started to notice them and that is how my panic was caused. Very unpleasant, nausea and shortness of breath. When the person in the app talked about it, it sometimes made it even worse for me.

P02; position 48-49

#### Theme 3: Side Effects

The theme of side effects covered codes addressing unwanted changes associated with the treatment but not related to the patients’ anxiety disorders. Side effects included simulation sickness (eg, “I got a little headache*”* [P02; position 36]) and the surfacing of other problems (eg, trauma), which were not addressed by the app:

I think there still is something. For now, I’d use the app, but through the treatment, other areas arose that were problematic, which were kind of hidden before. In that sense, you could say that it had a negative effect.

P10; position 44

#### Theme 4: Motivation Throughout the Treatment

Motivation was oftentimes addressed by the interviewed patients, depicting it as a dynamic process throughout the treatment. Many mentioned it as a crucial factor to the success or failure of their treatment. Different factors were given as reasons for an increase or decrease in motivation. Patients described the motivation to reduce symptoms to be able to master feared situations and increase well-being:

I was totally motivated, I wanted it, no matter what. For me it was so important, as from day to day, my life completely shifted and I couldn’t deal with it. Before, I was a happy and funny person and suddenly, I was afraid of everything.

P07; position 64

During the interviews, patients emphasized the necessity of being motivated for the, in large part, self-reliant treatment. Therefore, a lack of motivation was brought up as a potential risk factor for treatment success:

In the beginning [I was] very motivated, but at some point, that faded a little. And then I had a few weeks doing nothing. Then I realized I should do something and did a few exercises. But sometimes, I lack the motivation to sit down at home and engage [with the app]. I think it lacks the person that in some way “kicks your butt.”

P03; position 74

However, a high initial motivation was no guarantee for long-term engagement with the treatment app. For example, a patient reported a reduction in motivation to engage with the app due to a quick initial symptom reduction and overall improvement. Eventually, this made it harder to find the required time for the treatment or resulted in difficulties facing their anxiety in the frame of the treatment and a lack of translation into their everyday life:

In the beginning, it was fairly easy [to work with the app] because I knew it had to happen. The weeks before that weren’t really worth living. Once I got a grip on my anxiety, also because of the app and the therapy, it got harder, as it is quite time-consuming and I couldn’t estimate how long the next module would take.

P02; position 62

### Category 2: Mobile-Based Intervention–Related Themes

The second category covered 3 themes regarding the mobile-based app used in the intervention evaluated in this study: app content and exercises (theme 1), user interface (theme 2), and technicalities (theme 3). The displayed codes do not concern the mobile VRET, which is covered in another category (category 3: VRET-related themes).

#### Theme 1: Content and Exercises

The first theme covered patients’ opinions concerning the content and exercises of the app. Patients viewed the modules covering psychoeducation, the in-app push notifications, and the option to repeat contents as especially helpful:

I was surprised, how well the content was specialized to my panic disorder. I guess there is different content available that is shown to you, depending on what you have.

P02; position 62

Also, it was nice that I received notifications.

P04; position 33

Patients had mixed opinions on the relaxation exercises. Some described them as “extremely helpful” (P08; position 16-17), whereas others perceived them as rather misleading and distressing:

These breathing or mindfulness exercises partly worsened my panic. Well, definitely the breathing exercises, as I tried it a lot in the beginning. As soon as I gave room to my panic and tried to breathe against it, it became worse and made it take longer.

P02; position 34

Some patients expressed a desire for in-app contact with other patients or their psychotherapist to share progress or solve issues with peers:

It could be that for social anxiety, this isn’t realizable, but I could well imagine that it works like group therapy. So you could talk about it. “How was the situation for you?” “What was your take-home message?”

P11; position 144

#### Theme 2: User Interface

Overall, the app was perceived as well built regarding quality and user interface:

And I found [the app] qualitatively well-made. And it was good to listen to it and to read through it.

P11; position 96

The option to choose the in-app psychotherapist was brought up as an advantage. However, the importance of an option to speed up videos in the modules was highlighted. To further facilitate usability, a function to consume the content while being absent from the screen was desired:

Not being able to lock the screen, not being able to close the app. These are mechanisms that in some way make sense, but most of the time I used the app while cleaning, doing my laundry, or doing my homework. And then your cellphone is in another room and you have to click to proceed. That wasn’t severe, but also not comfortable.

P10; position 86

#### Theme 3: Technicalities

The sign-up process was marked as a problem source, and a transparent guide on how to obtain access to the treatment app and a function to download content were deemed helpful. However, it was also pointed out that technical difficulties, if they occurred, were quickly solved by the app support:

One time a situation didn’t load, so I contacted the support and they solved it fairly quickly, on the same day. It worked directly.

P11; position 88

### Category 3: VRET-Related Themes

The third category consisted of 3 themes covering opinions that were related to the mobile VRET modules of the treatment app. Participants discussed their experiences in the VRET, including the degree of immersion, the usability, and the variety of situations displayed on the treatment app.

#### Theme 1: Immersion

The first theme covered the perceived degree of immersion. Regarding this theme, several patients emphasized the feeling of immersion as helpful for their improvement and the ability of the app to create immersion and elicit fear:

And then I had anxiety, even though it was not real. It was quite surprising that I was able to do that. It was quite interesting and for some part even fun. Overall, I think it was mostly interesting to get to know more about [the anxiety].

P03; position 34

I think that is especially because I am conscious about being in a virtual situation. Other aspects, such as noises from the outside felt different. It is so advanced that you can completely exchange certain things, which somehow felt very real, without the real consequences, however.

P11; position 79

On the other hand, other patients found the prerecorded situations unrealistic, which could have negatively affected the degree of immersion. In that sense, it was addressed as a shortcoming that the mobile VRET consisted of 360° videos without possible interaction. This was perceived as especially problematic by patients with SAD as the prerecorded audience did not react to them:

You wear the VR-headset and you are sat in front of an audience, which is obviously filmed and the VR doesn’t really matter, because no matter what I do, they will always react the same. And that is not what causes my fear, it rather is the direct contact with others.

P01; position 26

So sometimes, when I misspoke, I had the feeling that it usually would’ve been a situation that would have caused a reaction. That took a bit of the immersion away. But I cannot estimate how much.

P11; position 82

In addition, some patients with PD or AG described a lack of anxiety when confronted with situations in mobile VR:

What I didn’t like, was the virtual [reality], yes, that didn’t match. I have to be honest, I sat on an airplane with weird people that did not look real and made repetitive movements. The airplane didn’t even shake or do anything that would have caused something in me. Or being alone in the basement. You would imagine this being creepy, yes? But nothing happened, not even a mouse hushed by or anything. I tried a lot, but that wasn’t for me. Or a shopping queue, nothing happened. Unrealistic. But for the rest, the app was great.

P07; position 28

#### Theme 2: Usability

The second theme was the usability of the VRET. While some patients did like facing feared situations at home, others felt discomfort when using mobile VRET:

My parents were home for the whole day so I could only train in situations when no one was at home. At some time in the evening, when I was sure that no one could hear me.

P05; position 66

Some patients described problems with the compatibility of the VR app and their smartphone despite their device meeting the manufacturer’s specified minimum technical requirements. For example, they mentioned lags in the 360° videos:

It should have worked with my phone, but it lagged so much, for five seconds it worked and then it stood still for another five.

P09; position 52

Regarding the VR headset, patients had problems with adequate fit and the glasses fogging up on the inside:

The VR headset did not really fit well and I got a bit of a headache because I wasn’t able to correctly adjust the resolution.

P02; position 36

Another patient brought up that the weight of the head-mounted construction with the smartphone inside made it difficult to wear the equipment for an extended period:

I found the headset very heavy. At some point, I had to put my elbows on my knees and by that point, it did not feel like not being at home on the couch.

P10; position 107-108

Finally, it was suggested that, as the app was only available for 90 days, the VR equipment should be only distributed temporarily as, when the license ends, the patient has no further use for the equipment that is tailored to the treatment app.

#### Theme 3: Variety of VR Content

A third theme covered the situations available in VR within the treatment app. Patients described the situations presented on the app as aligning well with fear-evoking situations in real life. They also liked the variety of agoraphobic situations they were able to experience without having to face these situations in real life:

So first of all, being able to identify the anxiety and then being able to directly test it. It was nice to directly go into the specific situations. So, thinking back, I would have never gone into the situation [in reality]. It was practical, I did not need to go somewhere to go through the fear.

P06; position 100

However, the situations were criticized for the lack of eventfulness. Patients did expect options to further increase fear and adjust situations:

I thought that the situations would increase in intensity. I thought that eventually, I would stand on a huge stage and give a speech. And I thought that something VR-related would happen with the people. Not being in a prerecorded situation, but something like a chat.

P11; position 100

While the app did implement situations for SAD (eg, giving a talk in front of an audience) and AG (eg, a bus ride), one patient criticized the lack of available situations for eliciting bodily arousal PD–related symptoms:

In the end, some modules did not fit my panic disorder anymore but fitted more to other anxiety disorders.... But I think that also was because I wasn’t able to pick a location that would cause my panic. For me, it is rather a personal, bodily thing. I tried one exercise on the airplane but after two minutes it was boring as it did not elicit panic.

P02; position 34-36 and position 62

### Category 4: Context-Related Themes

The fourth category comprised themes that addressed the framework in which the patients received the treatment.

#### Theme 1: Significance of Treatment Guidance

The treatment consisted of an app with mandatory psychotherapeutic sessions for the preparation and postprocessing of certain modules. Many of the patients addressed this guidance in their interviews. For most, this was a crucial ingredient for symptom improvement or a protective factor against deterioration. In addition, patients who did not particularly like the treatment app or the mobile VRET components still regarded the psychotherapeutic sessions as helpful:

The therapist definitely helped. Just being able to talk and also for my understanding. So, the empathy and to accept it as normal and not be upset about it.

P02; position 27-28

Despite the guidance, it was criticized that patients could not share the completed modules with their psychotherapist. However, the combination of the treatment app and the psychotherapeutic sessions was brought up as a well-rounded mix by most patients. One of the reasons given was that the appointments facilitated the integration of the content learned on the app:

After I held the speech [in VR] I had an appointment and the therapist was able to improve my anxiety a little, which I would have never achieved on my own. That’s why it was very good that I still had the appointments. Completely on my own, it wouldn’t have been possible.

P03; position 38

Some patients addressed the desire for more appointments accompanying the app-based treatment:

I would’ve appreciated it if the time between the appointments was shorter and that we would’ve met more often and spoken more.

P07; position 82

#### Theme 2: Study Schedule

Patients attended psychotherapeutic appointments at prearranged intervals as part of the RCTs. Most patients indicated not feeling like “being part of a research scenario” (P01; position 54) but being glad to contribute to research findings. However, to enable the manualized appointments in the experimental condition, the patients were asked to engage with the treatment app between the scheduled sessions. While this was perceived as helpful for staying motivated by most patients, some patients perceived it as stressful. The schedule was linked to treatment outcomes and limited the flexibility of the treatment:

First I felt relieved because usually, you have to wait a long time for a therapy spot. I was happy about any help, after all. It went quickly and I felt relieved, but also obligated to do this and that module between the appointments.... A downside was that time-wise, you are independent, but also in this study combination. If you have this time frame in which you have to finish certain modules before coming to the next appointment, it is a downside for me, as it limits my flexibility.

P02; position 13-14 and 56-57

#### Theme 3: Motives for Study Participation

In their interviews, patients expressed their motives for participating in this study. For example, one patient was motivated to improve the supply situation for women in mental health care:

It is super important to generate new findings, therefore I find [participating in research] always positive. Especially, I think for many diseases, women are underrepresented, and when I can contribute to improving this and technology.

P02; position 69-70

Another motive was receiving access to therapy as this is often hindered by long waiting times. Patients hoped to be prioritized by contributing to research:

I find the first step very hard and also exhausting when you are looking for a therapist. And when through [the study], you can quickly talk to someone, I consider this helpful, as you are rewarded for your first step and you don’t need to call another 20 [psychotherapists] and receive 19 rejections.

P01; position 62

#### Theme 4: Role of Mobile-Based Interventions in Mental Health Care

The fourth theme consisted of different opinions regarding the role of app treatment in mental health care. As such, IMIs were considered as an add-on to conventional therapy (eg, as an alternative to traditional homework or to bridge the waiting period before beginning psychotherapy):

Sometimes I think [waiting times] take way too long. The anxiety or the problems get more severe the longer it takes until they finally receive treatment. With this, you can at least work a bit on your own.

P07; position 198

In addition, IMIs were considered as having fewer barriers. This way, they could be a well-suited first step for patients searching for help:

I think many people are afraid to attend therapy in the first place or to completely trust another person. So I think it is easier to start the treatment with an app. For example, writing down things in the app without the therapist noticing. So I can imagine that this could lessen the barrier to starting a treatment.

P11; position 67

However, while IMIs were perceived as suitable for complementing therapy or bridging waiting times, some patients also described limitations in their applicability. They thought that IMIs might only be suited for certain aspects of treatment or patients. For example, some people might prefer books over using their smartphones to inform themselves about their disorders. P05 mentioned that she perceived the app treatment as helpful as she already knew what her problem was:

If you have problems in many areas of your life and the app asks you to name your problems and then you type down an eternally long text, in that case, a real conversation is more practical. However, if you know your problem and just want to work on it.... For me, that was very helpful and I think that could also be helpful for others.

P05; position 82

Patients consistently made clear that, from their experience, IMIs could be a complementary tool but should not replace psychotherapy:

Difficult. I think that depends on the person. But I cannot imagine [IMIs replacing therapy]. Not in the current state.

P03; position 96

## Discussion

### Principal Findings

#### Overview

The goal of this study was to systematically analyze patients’ perspectives concerning the integration of mobile VRET into guided IMIs for treating anxiety disorders. This was done to inform how patients perceive this innovative treatment approach and where chances and challenges might lie. This way, in 4 overarching categories, 14 themes were identified: symptom improvement, no change and deterioration, side effects, motivation, content and structure, user interface, technicalities, immersion, usability, variety of situations, treatment guidance, study schedule, motives for study participation, and role of IMIs in mental health care.

#### Perspectives on Treatment Outcomes

The first category of themes captured patients’ experiences with symptom alleviation throughout the treatment. Most striking about the themes in this category was that patients articulated a variety of reasons for their change or no change in symptoms and linked these to different aspects of the treatment. Improvement was explained by oftentimes highlighting the helpful interactions with the guiding psychotherapist but also by pointing out especially helpful treatment components such as psychoeducation, which was easy to follow as well as informational. Mobile VRET was also frequently brought up as allowing for the controlled encounter of feared and previously avoided situations [30]. Thus, fear responses can be elicited in a controlled virtual setting, enabling a confrontation of fears in line with the principles of in vivo exposure therapy, leveraging gradual exposure and habituation while maintaining the control of a simulated environment. By reducing logistical barriers, mobile VRET highlights the potential it holds in mental health care [35], which is further supported by meta-analytic findings yielding no inferiority of VRET compared to in vivo exposure therapy concerning primary treatment outcomes [61-63]. No change was explained by the treatment components not meeting the individual symptomatology, showing that the superficial modality of an app-based treatment might not fit every patient. The results give rise to the argument that treatment apps alone may not provide the depth of therapeutic interaction needed for comprehensive mental health care. Regarding the theme of side effects, how to deal with adverse events related to the treatment needs to be reflected on [23], as it happened to one patient, who learned through the treatment that there were other issues they needed to deal with for which there was no room in the manualized short-term treatment. Without the frame of the study context, this occurrence could have gone unnoticed, and the patient would most likely not have received further treatment.

Bringing together these findings with the results of previous quantitative studies, it can be stated that there are very few but promising studies on the efficacy of mobile VRET in IMIs. These emphasize clinical improvements and provide initial evidence of treatment benefits, particularly for components such as mobile VRET [46,64]. While additional quantitative studies have yet to profoundly support the treatment’s efficacy, the current qualitative studies can expand the findings by showing that the patient-perceived treatment outcomes are often tied to specific treatment components, such as psychoeducation, VRET, or tension regulation. These components have also been found efficacious in quantitative research [42,65], whereas other qualitative studies have determined motivation, symptom insight, and social support as crucial factors in treatments incorporating IMIs [52,53]. In comparison, the qualitative approach used in this study highlighted that the personally perceived commitment, motivation, and outcome expectancy were intertwined with personal impairments (anticipatory anxiety limiting daily routine and reinforcing the circle of avoidance and increase in fear over time) and treatment goals (building confidence for certain scenarios and gaining tools to manage physiological symptoms), which cannot be taken into account in many quantitative approaches.

Patients pointed out motivation as a central construct when addressing treatment outcomes. While motivation has also been found to be a key factor in traditional psychotherapy [66], it appears to play an even bigger role when the patient is self-reliant in working through most of the treatment content [67]. In particular, motivation was described as a dynamic process that is highest in the beginning, when the psychological strain is highest, and decreases throughout the treatment. After its decrease, it is then associated with no change or even deterioration, resembling previous findings on motivation in traditional psychotherapy [68]. For this treatment approach, this finding highlights the need for additional motivators as the treatment progresses to protect the patient from demotivation and losing benefits from the treatment.

#### Perspectives on the Treatment App

The second category of themes covered patient opinions regarding the treatment app. Most strikingly, psychoeducation was brought up as an overwhelmingly positive treatment component that can be delivered well via an app, which is in line with previous research on patient perspectives on digital treatment modalities [69]. In addition, a high degree of individualization (eg, choosing the voice of the in-app psychotherapist) and an adjustable user interface were stressed as preferable. An aspect that faced repeated criticism was the in-app relaxation. While mindfulness interventions have been found efficacious in mental health care [70], this sample encompassed patients with anxiety disorders. It might be that, for such disorders, in particular regarding PD, relaxation is better delivered via psychotherapeutic interaction than through an app considering the possibility of relaxation-induced panic attacks [71]. It is also known that confrontation with situations and contexts that have previously been avoided contributes to an initial increase in fear or discomfort, which is positively associated with treatment success [72]. However, in the framework of self-guided or blended treatment, patients might be more inclined to discontinue treatment as they perceive the initial negative emotional states as threatening, thereby reinforcing avoidance behavior and, ultimately, intensifying the risk of unwanted events. For patients, it was also important to have quick access to the treatment app and be able to use it without barriers. One of these barriers was long informational sections, which patients could not speed up or listen to while being active (eg, walking or cleaning) as the app would require the user to click to move along. These findings are comparable to those of other qualitative studies on opinions on app treatment as, in general, a desire for a high degree of personalization stood out in this study as well [69]. However, while implementing the patients’ suggestions might lead to more treatment acceptance, whether these suggestions could raise other issues has to be carefully considered for each suggestion on its own. For example, allowing for the locking of the screen or opening of other apps while using the treatment app could lead to less focus on the treatment and, eventually, a decline in motivation or less treatment success.

#### Perspectives on Mobile VRET

In the third category, themes were listed that were related to the mobile VRET of the treatment app. The most discussed aspect was the degree to which the head-mounted construction with the patient’s smartphone serving as a display was able to create an immersive experience for VRET [43]. While it was welcomed by some patients, who described being pleased with the high immersion, others criticized a lack of interaction given the fact that the mobile VRET consisted of prerecorded 360° videos. As previously outlined, different systems are used in VRET that are either equipped with dynamic interaction options or, as in this study, based on 360° videos that do not offer the chance to affect the scenarios or characters in them. Patients addressed this by pointing out a lack of interpersonal interaction during VRET, which made the experience less immersive. This aligns with the clinical presentation of SAD, where interpersonal interaction plays a central role [5,44], and suggests that the degree of the immersive interaction required might vary depending on the disorder. Nonetheless, many participants across all 3 anxiety disorders found the 360° videos personally beneficial for treating their anxiety, appreciating the practical benefits of accessibility and ease of use, supporting their engagement during the exposure tasks. It is important to note that these evaluations were conducted without directly comparing 360° videos to interactive VRET systems, which could be addressed in future research. In this study, the hardware was identified as a problem source in the theme of usability. These findings suggest that mobile VRET still needs time to overcome these particular challenges but, overall, can be useful in treating SAD, PD, and AG.

Although numerous studies have drawn out the possibilities of VRET in mental health care [35], it is yet to be seen how the intriguing treatment approach of using mobile VRET can be further improved to be well received by patients. One way could be to integrate desired elements such as the possibility to choose from a large pool of fear-evoking situations with optional adjustments, such as the crowdedness of certain places or the pace of car, bus, or tram rides, as brought up by the interviewed patients.

#### Perspectives on the Study’s Context

Themes in the fourth category, which were context related, showed that, for the patients, the psychotherapeutic supervision was very important, the study schedule may have had an influence on symptom change, and most patients could well imagine using (guided) treatment apps but could not imagine them as a substitute for psychotherapy. Overall, the interviewed patients highly valued the psychotherapeutic sessions as part of the guided treatment approach. Despite the app being the main ingredient of their treatment, the psychotherapeutic guidance was mentioned in all interviews and was always brought up positively or with the request to have more psychotherapeutic sessions. This finding is in line with those of previous literature that found guided IMIs to be superior to unguided ones [26], indicating that alliance as a mechanism of change also holds up for digitally delivered interventions [73]. While the general modality of using IMIs is partly aimed at reducing psychotherapeutic involvement [23], how far this reduction is taken should be carefully considered for each treatment approach. In this study, many interviewed patients distinguished the psychotherapeutic appointment as the most remarkable factor in their treatment, whereas conceptually, one could argue that it was only intended as a supporting element, with the main focus laying on the treatment app with mobile VRET [47,48].

The thematic connection between the theme of study schedule and the theme of symptom improvement originated in patients giving aspects of the study schedule as reasons for their improvement. For example, it was mentioned that the psychotherapeutic appointments and calls from the study team served as deadlines and reminders to finish the modules in the treatment app, which then led to a more holistic understanding of etiological and maintaining factors underlying their anxiety disorder. Here, it is important to note that these checkups would not have occurred in a real clinical setting. Despite making sure that the RCTs from which the interviewed patients were recruited accurately reflected real-world clinical settings, it is essential to acknowledge that the patients still engaged in a structured intervention within a controlled environment [47,48]. Therefore, these minimal but necessary alterations could have influenced the quantitative results. Beyond trials investigating the treatment efficacy, it is important to also further identify characteristics in patients (eg, demographic and clinical variables, personality traits, and coping mechanisms), as well as process (eg, fear reduction during exposure) and context variables that may predict the treatment outcomes of these interventions and develop clear indications as to who should gain access to these treatment options [72,74,75]. In addition, for this reason, future interventions could increasingly incorporate deadlines and checkups to increase the patients’ commitment to engage with the otherwise self-reliant treatment contents.

While treatment acceptance is a crucial factor in mostly self-delivered treatments, in their interviews, patients also reflected on the role of IMIs in the mental health care system. Due to the scarcity of treatment capabilities, they could imagine IMIs as a way to bridge waiting times. However, they could not imagine the tested treatment substituting psychotherapy as a whole, which is comparable to other qualitative research on patient attitudes toward IMIs [54]. This finding is also in line with those of research on the perspective of psychotherapists on IMIs, who welcomed the digital treatment option as an aid for certain aspects but also could not imagine it as a substitute for psychotherapy [76].

Relating this study’s results to the current situation of outpatient psychotherapy, several advantages and challenges of the investigated treatment modality can be highlighted. As accessibility and a low threshold for engaging with IMIs were appreciated, they might be advantageous in bridging waiting times or reducing stigma or hesitancy associated with seeking psychotherapeutic treatment [26]. This might be especially beneficial in countries with long waiting times, such as Germany [77]. Another advantage was that the treatment functioned as digital homework, contributing to problem activation outside the treatment, which has been associated with treatment success in the past [18]. Patients also listed the cost and scalability of the treatment as potential advantages over outpatient psychotherapy. However, oftentimes, the importance of psychotherapeutic guidance was emphasized, which is why a challenge might lie in the self-guided nature of mobile VRET. Namely, the procedure partly opposes current treatment guidelines [10], indicating that initial exposure attempts are best psychotherapeutically accompanied to prevent adverse effects or misapplication (ie, premature termination). In addition, a lack of individualized support remains a challenge, which was criticized by patients in direct comparison to regular outpatient psychotherapy.

### Limitations and Future Research

While this study was capable of drawing a multifaceted picture of patients’ opinions, some limitations need to be addressed when interpreting the findings. First, the findings of this study are derived from a blended IMI with integrated mobile VRET, indicating that the observed benefits or challenges may not directly translate to other treatment modalities. For instance, experiences with unguided IMIs or stationary VRET may differ from those with blended IMIs with mobile VRET. In addition, while the reliance on a qualitative method of data analysis to interpret the obtained interview transcripts offers rich insights, it also inherently requires caution when interpreting causality. Such an approach captures patient perspectives but does not allow for claims regarding the treatment’s efficacy, which would require further exploration through controlled, comparative, and quantitative research designs. Future trials of a confirmatory nature are needed to further investigate the exploratory findings of this study.

Another point worth noting is that the interviews were conducted as part of a larger RCT. From the patients’ perspective, it was interpreted that the study schedule, despite being incorporated into an outpatient clinical setting, might be linked to the obtained results. Differences between the study and treatment as usual include, for example, a high degree of standardization, required comparability of therapeutic settings, frequency and duration of therapy, and a reliance on treatment manuals. Therefore, for future research, it could be beneficial to conduct studies in a more naturalistic setting. This could include investigating treatment adherence and feasibility in less structured clinical settings such as standard outpatient care. Another relevant option for future research could be investigating challenges to the treatment’s accessibility in contexts with different resources and infrastructure (eg, rural vs urban regions) as IMIs and mobile VRET in particular are theorized to possibly address disparities in the supply of mental health care by reducing geographical barriers and providing scalable solutions in underserved areas.

### Conclusions

This study explored patients’ perspectives on IMIs embedding mobile VRET for SAD, PD, and AG. As such, various components such as the psychoeducational content were listed as beneficial in the intervention, and motivation was continuously perceived as a crucial factor for treatment engagement. The mobile VRET was welcomed as an innovative add-on to overcome anxiety but, for some patients, lacked immersion and personal fit due to the reliance on prerecorded 360° videos without interaction. Finally, the psychotherapeutic guidance of the intervention was appreciated as especially beneficial and stood out as a central aspect in most of the interviews as it helped the patients integrate their learning from the IMI and served as a deadline to engage with the IMI before the next treatment session. This study has shown that, in the further development of IMIs and other associated interventions such as VRET, it is important not only to empirically test the efficacy of such procedures or their acceptance among psychotherapists but also not to ignore the perspectives of patients and those affected as these are highly valuable to further improve existing procedures as well as ensure their applicability. For future research, it is suggested to explore patients’ perspectives on other treatment modalities or investigate the findings of this study using confirmatory research designs.

## Abbreviations

AG: agoraphobia
CBT: cognitive behavioral therapy
IMI: internet- and mobile-based intervention
PD: panic disorder
RCT: randomized controlled trial
SAD: social anxiety disorder
VR: virtual reality
VRET: virtual reality exposure therapy
